# Artificial intelligence for the diagnosis of heart failure

**DOI:** 10.1038/s41746-020-0261-3

**Published:** 2020-04-08

**Authors:** Dong-Ju Choi, Jin Joo Park, Taqdir Ali, Sungyoung Lee

**Affiliations:** 1 Division of Cardiology, Department of Internal Medicine, Seoul National University Bundang Hospital, Seongnam, Republic of Korea; 2 Department of Computer Science and Engineering, Kyung Hee University, Yongin, Republic of Korea

**Keywords:** Outcomes research, Heart failure

## Abstract

The diagnosis of heart failure can be difficult, even for heart failure specialists. Artificial Intelligence-Clinical Decision Support System (AI-CDSS) has the potential to assist physicians in heart failure diagnosis. The aim of this work was to evaluate the diagnostic accuracy of an AI-CDSS for heart failure. AI-CDSS for cardiology was developed with a hybrid (expert-driven and machine-learning-driven) approach of knowledge acquisition to evolve the knowledge base with heart failure diagnosis. A retrospective cohort of 1198 patients with and without heart failure was used for the development of AI-CDSS (training dataset, *n* = 600) and to test the performance (test dataset, *n* = 598). A prospective clinical pilot study of 97 patients with dyspnea was used to assess the diagnostic accuracy of AI-CDSS compared with that of non-heart failure specialists. The concordance rate between AI-CDSS and heart failure specialists was evaluated. In retrospective cohort, the concordance rate was 98.3% in the test dataset. The concordance rate for patients with heart failure with reduced ejection fraction, heart failure with mid-range ejection fraction, heart failure with preserved ejection fraction, and no heart failure was 100%, 100%, 99.6%, and 91.7%, respectively. In a prospective pilot study of 97 patients presenting with dyspnea to the outpatient clinic, 44% had heart failure. The concordance rate between AI-CDSS and heart failure specialists was 98%, whereas that between non-heart failure specialists and heart failure specialists was 76%. In conclusion, AI-CDSS showed a high diagnostic accuracy for heart failure. Therefore, AI-CDSS may be useful for the diagnosis of heart failure, especially when heart failure specialists are not available.

## Introduction

The prevalence of heart failure (HF) has been increasing^1,2^. HF is associated with high morbidity and mortality^3^. Because HF is a complex syndrome that can result from structural and functional cardiac disorder, rather than a single disease entity, its correct diagnosis can be challenging even for HF specialists. Currently, HF is classified according to ejection fraction, i.e., HF with reduced ejection fraction (HFrEF), HF with mid-range ejection fraction (HFmrEF), and HF with preserved ejection fraction (HFpEF)^4^. A correct diagnosis is mandatory before proper treatment can be initiated^4,5^. Furthermore, present-day physicians are challenged by rapidly changing scientific evidences, new drugs, and the complexity of guidelines for HF management, especially in outpatient clinic. With enormous advancements in information and communication technologies, such as easy storage, acquisition, and recovery of big data and knowledge, artificial intelligence (AI) has been gaining an important role in cardiology^6^.

Two types of AI decision systems are available: a white-box-based and a black-box-based one. A white-box AI-based decision system involves correlations and transparency among rules for the analysis of accumulated data, and is mainly constructed using supervised algorithms such as decision tree algorithm^7^. On the contrary, a black-box-based AI has opaque algorithms and its process and reasoning applied in providing the respective conclusions are difficult to clarify. IBM Watson for Oncology (WFO) is black-box AI-based decision systems. WFO demonstrated a concordance rate of 93% for the treatment recommendation in breast cancer. However, WFO cannot disclose the recommendation processes for the final clinical decision^8^.

Clinical Decision Support System (CDSS) is a health information technology that assists physicians in clinical decision making. The concept of computer-based clinical decision has been developed for informatics six decades ago^9^. In spite of the enthusiasm for evolving CDSS which is assisted with the potential of AI, the realities and complexities of real clinical practice limit the rapid evolution of CDSS. An effective CDSS requires CDSS to match the individual patient’s characteristics to the clinical knowledge base, provides patient-centric assessments and recommendations, and finally presents recommendations in white-box manner to the physicians for their final decision^8^.

We conducted a study assessing the level of agreement with respect to the HF diagnosis to identify the three types of HF, i.e. HFrEF, HFmrEF, and HFpEF, between HF specialists and AI-CDSS at a tertiary center in Korea. First, we created an AI-CDSS using a hybrid approach of expert-driven knowledge acquisition and ML-driven rule generation. Second, we evaluated the diagnosis concordance (degree of agreement) of AI-CDSS in a test set of patients with and without HF as a pilot clinical study. Third, we prospectively tested the diagnostic performance of AI-CDSS in consecutive patients presenting with dyspnea to the outpatient clinic.

## Results

### Development of cardiovascular AI-CDSS

Using the training dataset of 600 patients with and without HF, the AI-CDSS was created using predefined steps including expert-driven knowledge acquisition, machine learning (ML)-driven rule generation, and hybridization of both types of knowledge.

#### Expert-driven knowledge acquisition

In the knowledge modeling phase, the clinical recommendations of diagnosis were first transformed into mind maps and then transformed to a decision tree. The decision tree was evaluated and modified by the physicians until a consensus was achieved. The final decision tree was termed as R-CKM (Supplementary Fig. 1) and included 14 contributing factors (Supplementary Table 1) and 4 possible outcomes: HFrEF, HFmrEF, HFpEF, and no-HF.

#### ML-driven rule generation

We used five machine learning algorithms i.e. Decision Tree (DT), Random Forest, Chi-squared Automatic Interaction Detection (CHAID), J48, and Classification and Regression Tree (CART). All algorithms selected only few features such as left ventricular ejection fraction (LVEF), left atrial volume index (LAVI), and left ventricular mass index (LVMI) as highly contributing factors (Supplementary Table 2). To boost the model performance, the auto-feature selection method was used and LVEF, electrocardiography, LVMI, and LAVI were selected as the most significant features (Supplementary Fig. 2).

The five algorithms showed different accuracy (Supplementary Table 3). We also calculated the rank of each algorithm based on the accuracy, number of rules extracted, and number of attributes involved, using the rank formula developed in our previous work^10^. Finally, CART algorithm was selected to create ML-driven knowledge, because it showed the highest accuracy and rank of 88.5 and 0.5736, respectively. The CART algorithm mainly focused on features of LVEF, LAVI, and tricuspid regurgitation velocity (Supplementary Fig. 3). The algorithm correctly predicted HFmrEF and HFrEF with 100% accuracy, whereas HFpEF and no-HF were predicted with 78.9% and 80.5% accuracy, respectively.

#### Hybrid knowledge

**Fig. 1:**
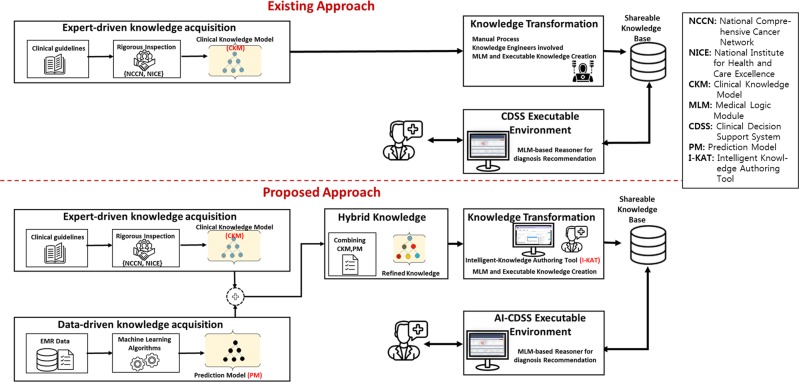
Comparison of existing CDSSs and our proposed artificial intelligence-CDSS. CDSS Clinical Decision Support System, CKM clinical knowledge model, I-KAT Intelligent Knowledge Authoring Tool, NCCN National Comprehensive Cancer Network, NICE National Institute for Health and Care Excellence, PM prediction model.

The merging of the CKM from the expert-driven knowledge and the PM from the ML-driven knowledge approach led to the final hybrid knowledge in form of R-CKM (Fig. 1, Supplementary Materials). Sometimes, physician may miss some of the attributes or path of attributes during development of CKM, and the ML generated PM finds the missing attributes or paths. For instance, the CKM is starting with the “Sign & Symptoms” as shown in (Supplementary Fig. 3), while the PM starts checking from “LVEF” as shown in (Supplementary Fig. 4). Therefore, the hybridization algorithm recognizes that the CKM is missing a path of “Not Available” values between “Sign & Symptoms” and “LVEF” attributes. When we added this new path into CKM, the number of knowledge base rules increased drastically. The addition of new path into R-CKM increases the coverage of patient cases to generate right recommendations and increase the accuracy.

### Validation of AI-CDSS

#### Study population

The test dataset included 598 patients (490 patients with HF, 108 patients without HF). Patients with HF were older (73.1 ± 13.8 years vs. 64.8 ± 13.8 years, *P* < 0.001), more likely to be male (52% vs. 37%, *P* = 0.005), and had higher N-terminal pro-brain natriuretic peptide levels (10,075 ± 11,778 pg/L vs. 82 ± 68 pg/L, *P* < 0.001). Concerning the echocardiographic parameters, patients with HF had lower LVEF (45.5 ± 17.4% vs. 64.1 ± 6.5%, *P* < 0.001), higher LAVI (53.9 ± 21.1 ml/m^2^ vs. 31.2 ± 8.5 ml/m2, *P* < 0.001), and higher E/e′ (18.6 ± 9.8 vs. 9.8 ± 3.5, *P* < 0.001) (Table 1). Among patients with HF, 199 (40.6%), 63 (12.9%), and 228 (46.5%) were classified as having HFrEF, HFmrEF, and HFpEF, respectively.

**Table 1:** Characteristics of the study population retrospective patients (*n* = 598).

	No heart failure	Heart failure				*P* value^*^
		All	HFrEF	HFmrEF	HFpEF	
Age (years)	64.8 ± 13.8	73.1 ± 13.8	70.3 ± 14.6	74.7 ± 14.1	75.2 ± 10.6	<0.001
Male (%)	37	52	54.3	52.4	50.0	0.005
HF symptoms, signs (%)	81.5	89.8	94.0	84.1	87.7	0.015
Clinical history (%)	14.8	51.6	66.3	55.6	37.7	<0.001
Physical exam (%)	9.3	51.4	60.8	49.2	43.9	<0.001
Abnormal ECG (%)	46.0	93	99.5	96.8	86.2	<0.001
NT-pro-BNP (pg/L)	82.4 ± 68.0	10075 ± 11778	15665 ± 12604	8634 ± 9666	5595 ± 9306	<0.001
Echocardiography						
LVEF (%)	64.1 ± 6.5	45.5 ± 17.4	27.1 ± 7.5	45.3 ± 2.6	61.6 ± 6.5	<0.001
LAVI (mL/m^2^)	31.2 ± 8.5	53.9 ± 21.1	60.5 ± 18.6	52.6 ± 27.5	48.0 ± 19.4	<0.001
LVMI (mg/m^2^)	83.4 ± 18.3	127.3 ± 44.7	151.0 ± 41.5	129.0 ± 50.7	106.0 ± 33.9	<0.001
*E*/*e*	9.8 ± 3.5	18.6 ± 9.8	22.9 ± 10.34	17.4 ± 8.6	15.6 ± 8.3	<0.001
Septal e′ (cm/s)	6.9 ± 2.4	5.0 ± 2.1	4.2 ± 1.7	5.1 ± 2.3	5.6 ± 2.2	<0.001
TRV (m/s)	2.6 ± 1.5	2.9 ± 0.7	3.0 ± 0.7	2.8 ± 0.5	2.8 ± 0.7	0.001
GLS (*n* = 324) (%)	16.4 ± 3.9	10.8 ± 5.0	7.1 ± 2.7	10.4 ± 2.8	14.6 ± 4.3	<0.001

^*^P value between no heart failure and heart failure.

*ECG* electrocardiography, *GLS* global longitudinal strain, *HF* heart failure, *LAVI* left atrial volume index, *LVEF* left ventricular ejection fraction, *NT-proBNP* N-terminal pro-B-type natriuretic peptide, *TRV* tricuspid regurgitation velocity.

#### Diagnostic accuracy

**Fig. 2:**
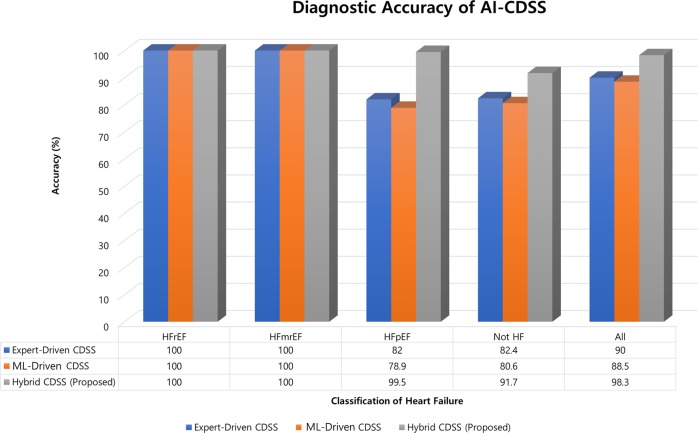
Comparative analysis of the diagnostic accuracy of different approaches in the retrospective cohort. CDSS Clinical Decision Support System, HFmrEF heart failure with mid-range ejection fraction, HFpEF heart failure with preserved ejection fraction, HFrEF heart failure with reduced ejection fraction.

The results of comparative analysis are shown in Fig. 2. The concordance rate was 100% in HFrEF and HFmrEF for all three approaches. With respect to HFpEF, the concordance rate was 82%, 79%, and 99.5% for expert-driven, ML-driven, and hybrid CDSS, respectively. Similar findings were observed for no-HF. The overall diagnostic accuracy was 90%, 88.5%, and 98.3% for expert-driven, ML-driven, and hybrid CDSS, respectively, showing a remarkable increase in accuracy by 8% with the hybrid approach, i.e., AI-CDSS.

The expert-driven approach had a sensitivity and a specificity of 0.96 and 0.71, respectively (Supplementary Table 4), whereas the ML-driven approach had a sensitivity and a specificity of 0.72 and 0.94, respectively (Supplementary Table 5). Strikingly, the hybrid approach had a sensitivity and specificity of 0.94 and 0.99, respectively (Supplementary Table 6).

#### Subgroup analysis

We divided the patients according to echocardiographic parameters. Set A included all echocardiography parameters, whereas set B included only LVEF, LAVI, and LVMI. The concordance rate was lower in set B than in set A (Supplementary Fig. 4). In our study, the age of the included patients ranged from 20 to 92 years. Age did not affect the accuracy of the system (Supplementary Table 7).

### Accuracy of AI-CDSS in a prospective cohort of patients with dyspnea

**Fig. 3:**
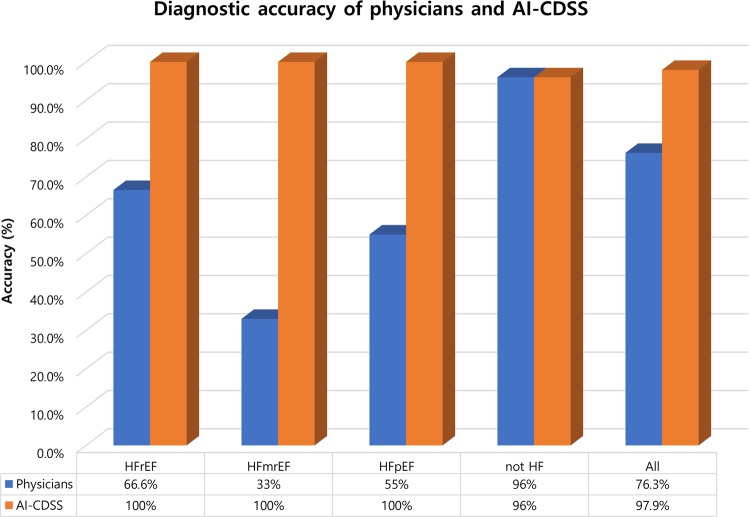
Comparative analysis of the diagnostic accuracy of physicians and AI-CDSS in the prospective cohort. Abbreviations are as in Fig. 2.

A total of 100 consecutive patients who presented with dyspnea to the outpatient clinic were enrolled. Of these, the data of three patients were not complete; thus, the data of 97 patients were used in the final analysis. Of the 97 patients, 43 (44%) had HF. In this prospective cohort, the concordance rate of the non-HF specialists was 76%, whereas that of AI-CDSS was 98% (Fig. 3). Especially, the diagnosis of HFmrEF and HFpEF was low among the non-HF specialist, whereas the diagnosis of no-HF was comparably high.

## Discussion

Correct diagnosis of HF can be challenging for physicians, even for HF specialists. In this study, we first created AI-CDSS by using the data of 1198 patients with and without HF and showed that AI-CDSS had very high diagnostic accuracy in these patients. In a prospective cohort of patients presenting with dyspnea to the outpatient clinic, AI-CDSS consistently showed a remarkably high diagnostic accuracy. By contrast, non-HF specialists showed a relatively low diagnostic accuracy for HF. Therefore, AI-CDSS may be useful for the diagnosis of HF, especially when HF specialists are not available.

CDSS has been applied in clinical diagnosis^11^, preventive care^12^, and chronic disease management^13^, among others. It provides a proficient decision-making service to improve the quality of healthcare, but has several complexities and limitations^8^. Generally, AI-CDSS acquires knowledge from structured and unstructured data using ML and natural language processing techniques^14,15^. The amalgamation of ML-driven rule generation with expert-driven knowledge acquisition enhances the system accuracy^10^. Therefore, we chose the hybrid approach for the knowledge acquisition of AI-CDSS, which includes three distinct steps: expert-driven knowledge acquisition, ML-driven rule generation, and hybridization of both types of knowledge.

In expert-driven knowledge acquisition, we first built CKM by transforming expert-driven knowledge into a mind map and decision tree^10^. Finally, the decision tree was validated with the PM of ML-driven knowledge.

In ML-driven knowledge acquisition, we created a PM with an available big dataset. Various ML algorithms can be used to analyze and extract the hidden patterns in the form of knowledge models. In our study, we used white box AI and causal machine algorithms such as decision tree, random forest, CHAID (Chi-squared Automatic Interaction Detector), J48, and CART/CRT. White-box model AI are selected for their transparency, which enables easily determining all attributes for classification and verification of new patient data^16^. They also increase the physicians’ satisfaction level, because the rationale for a decision is also provided to the physician using the features contributing to the final decision. In addition, the computational complexity of the decision tree constructing algorithms (white box) is relatively low. By contrast, black box algorithms have no transparency in knowledge modeling owing to difficulty in interpreting the inner working layers of the models.

In the hybridization of the both knowledge types, we validated the PM from the ML-driven approach against the mind map of CKM from the expert-driven approach to produce the final hybrid knowledge in form of R-CKM.

Finally, we developed a web-based application in the form of cardiovascular AI-CDSS for use of physicians in real clinical practice. For this purpose, the R-CKM knowledge was transformed into MLM for knowledge shareability and computer-executable format using I-KAT, which had been developed by our team^7^. The resultant knowledge can be easily shared and integrated into various formats of HF diagnosis systems, because the resultant knowledge was built with consolidation of the standard data model vMR (Virtual Medical Record) and the standard terminology SNOMED CT (Systematized Nomenclature of Medicine—Clinical Terms).

Because HF is a syndrome with various clinical features, its diagnosis can be very challenging even for HF specialists. In patients with HF, pulmonary congestion can develop because of congestion in the left heart, causing dyspnea. However, dyspnea as a symptom can also arise from lung disease, anemia, and mental disorders^17^. Leg swelling is a typical sign of congestion in the right heart. However, it also has many differential diagnoses, including kidney disease, adverse effect of drugs, and chronic venous insufficiency, among others^18^. In clinical practice, many patients are diagnosed as having HF even if they do not have HF, and vice versa. A correct diagnosis of HF is crucial because patients with HF have a grave prognosis that is comparable to that of oncologic malignancies^19^, and there exist therapy that can improve survival in patients with HF^4,6^. Consequently, misdiagnosis of HF can hinder the chance of improving the outcomes. AI-CDSS is a tool that helps in making better medical decisions, thereby reducing clinical errors and improving the quality of life. It has the potential to generate alerts and reminders, diagnostic assistance, therapy critiquing and planning, and image recognition and interpretation.

Currently, HF is classified according to LVEF into HFrEF, HFmrEF, and HFpEF. With respect to HFrEF, a decrease in LVEF may alert physicians to the possible diagnosis of HF. By contrast, for HFpEF >50%, the normal systolic function may “blind” the physicians and HFpEF may remain undiagnosed. We showed that AI-CDSS showed acceptably high concordance for diagnosing HF regardless of type, whereas non-HF specialists misdiagnosed HFpEF in almost half of the patients.

In medicine, IBM WFO demonstrated high concordance with oncologists in treatment recommendations^14^. In the field of cardiology, our study presents the clinical feasibility of AI for diagnosing HF.

There are several limitations in this study. The intervention of the physicians is crucial in knowledge creation and validation. However, the level of expertise varies from physician to physician, so that the CKM developed by physicians in a hospital may differ from that developed in another hospital. Similarly, because the attributes in the PM depend on the patient data used, they may also differ from variables recommended in the guidelines. Therefore, further studies are necessary to validate the AI-CDSS in other study populations.

In conclusions, AI-CDSS showed high diagnostic accuracy for HF, independent of HF types. Therefore, AI-CDSS may be useful for the diagnosis of HF, especially when HF specialists are not available.

## Methods

### Study population and data collection

#### Retrospective cohort

We included 1198 patients with and without HF from January 2016 to December 2017. We divided the patients into two datasets. The first 600 patients were used for the generation of AI-CDSS as a training dataset for ML, whereas the remaining 598 patients were used for the validation of the AI-CDSS as the test dataset. HF was defined as present when patients had symptoms (dyspnea, orthopnea) or signs of HF (rales, pitting edema, ascites) and met one of the following criteria: lung congestion, objective findings of left ventricular systolic dysfunction, or structural heart disease. Clinical information including demographics, symptoms, signs, medical history, laboratory examination, electrocardiography, and echocardiography was obtained. Control patients, i.e., those without HF, were randomly selected from the electronic medical records.

#### Prospective pilot cohort

For an additional validation of AI-CDSS, we enrolled 100 consecutive patients presenting with dyspnea to the outpatient clinic. The treating physicians performed history taking and physical examination, ordered diagnostic tests, and made a final diagnosis, i.e., HF or no-HF, according to their clinical judgment. The data were first recorded in an electronic clinical research form, and then automatically transferred to the AI-CDSS. A direct extraction of patients’ data from electronic medical record was circumvented because of the information security regulation at our institution.

The study protocol was approved by the institutional review board of the Seoul National University Bundang Hospital. For the retrospective cohort, the requirement for written informed consent was waived by the institutional review board. Each patient in the prospective cohort provided informed consent before study enrollment. The study complied with the Declaration of Helsinki.

### Echocardiography

All images were obtained using a standard ultrasound machine with a 2.5-MHz probe. Standard techniques were used to obtain M-mode, two-dimensional, and Doppler measurements in accordance with the American Society of Echocardiography guidelines^20^. Tissue-Doppler-derived peak systolic, early, and late diastolic velocities of the septal mitral annulus were recorded. Left ventricular end-systolic and end-diastolic volumes were measured from apical four- and two-chamber views and LVEF was calculated using Simpson’s biplane method.

### Generation of cardiovascular AI-CDSS

The traditional CDSSs usually focus on the expert-driven approach with collaboration between physicians and knowledge engineers, where the knowledge engineer is an expert in AI language who investigate the underlying problems, develop the main concepts, and efficiently represent the knowledge in the domain. The fundamental knowledge resource is the clinical practice guidelines and physicians’ expertise. The AI-CDSS uses patient data as the second important resource of knowledge after processing with ML algorithm (ML-driven approach). Figure 1 shows the difference between the traditional CDSSs and the cardiovascular AI-CDSS. The existing CDSSs maintain the knowledge base by the knowledge engineers. In contrast, AI-CDSS focuses on the hybrid approach of expert-driven knowledge acquisition and ML-driven rule generation and overcomes the physicians’ dependency on knowledge engineers. In AI-CDSS, the clinical knowledge model (CKM), a classical top-down decision tree, is generated by domain expert (physician) using guidelines and their experiences; it is called the Expert-Driven Knowledge. The second step is to create ML-based prediction model (PM) using several ML algorithms, which is called the machine learning (ML)-driven knowledge. The third step is to generate the refined-CKM (R-CKM) by the computer scientists using a quick, simple, and iterative agile software development. The R-CKM generation is composed of making prediction model of ML-driven knowledge and validation of expert-driven knowledge with respective ML-driven knowledge using several ML algorithms with training dataset of 600 patients’ data; it is called the hybrid knowledge. Finally, the R-CKM knowledge is transformed into shareable and interoperable setting in the form of Health Level-7 (HL7) complaint standard knowledge representation, termed Medical Logic Module (MLM), using the Intelligent Knowledge Authoring Tool (I-KAT) developed by our group. The executable MLM in the shareable knowledge base is executed to generate decisions based on the patients’ input to assist the physicians. More details of cardiovascular AI-CDSS are explained in the Supplementary methods.

### Study variables

HF was defined when patients had signs or symptoms of HF and either lung congestion, objective findings of LV systolic dysfunction, or structural heart disease. The diagnosis of HF was confirmed by two independent HF specialists who had >10 years of clinical experience. The diagnosis by the experts was considered the gold standard.

According to the LVEF on echocardiography, patients were classified as having HFrEF (LVEF < 40%), HFmrEF (40% ≤ LVEF < 50%), and HFpEF (LVEF ≥ 50%).

The diagnostic accuracy of AI-CDSS was measured using experts’ diagnosis as the gold standard. Concordance was defined as present when experts and AI-CDSS had the same diagnosis, i.e., both HF or both no-HF. Discordance was defined to exist when there was a disagreement between diagnoses.

### Statistical analysis

Descriptive statistics were calculated to determine the clinical characteristics and outcomes of the registry population. Data were presented as numbers and frequencies for categorical variables and as mean ± standard deviation or median with interquartile range for continuous variables. For the comparison between groups, the *χ*^2^ test (or Fisher’s exact test when any expected cell count was <5 for a 2 × 2 table) was used for categorical variables, whereas unpaired Student’s *t*-test was used for continuous variables. Concordance was expressed as the percentage agreement. Pearson’s correlation was used to calculate the association between expert opinion and AI-CDSS judgment.

A two-sided *P* value of <0.05 was considered statistically significant. Statistical tests were performed using IBM SPSS Statistics version 23 (SPSS Inc., Chicago, IL, USA).

### Role of the funding source

The funder of the study had no role in study design, data collection, data analysis, data interpretation, or writing of the report. The corresponding author had full access to all the data in the study and had final responsibility for the decision to submit for publication.

### Reporting summary

Further information on experimental design is available in the Nature Research Reporting Summary linked to this article.

## Supplementary information

Supplementary Information

Reporting Summary

## Data Availability

The dataset generated during the current study is not publicly available due to restrictions in the ethical permit, but may be available from the corresponding author on reasonable request.

## Code availability

We have made all source code available at https://github.com/ubiquitous-computing-lab/AI-CDSS-Cardiovascular-Silo.
