# Supplementary material for: Artificial intelligence for the diagnosis of heart failure
Source: NPJ Digit Med. 2020 Apr 8;3:54. doi: 10.1038/s41746-020-0261-3 (PMC7142093; doi:10.1038/s41746-020-0261-3)
Supplement: Supplementary file 1 — Supplementary Information [file 41746_2020_261_MOESM1_ESM.pdf]

Supplementary Materials

Supplementary Methods

Hybrid knowledge acquisition for cardiovascular artificial intelligence-Clinical Decision Support System (AI-CDSS)

The proposed system, AI-CDSS, for heart failure (HF) diagnosis evolves the knowledge base with the hybrid knowledge acquisition approach by using expert-driven and ML-drivenn approaches. **Figure 1** shows the complete methodology of knowledge evolution and execution to provide recommendations for HF diagnosis. The knowledge acquisition methodology is inspired from our previous works.[13,15]

Expert-driven knowledge acquisition
In expert-driven knowledge acquisition, we acquired knowledge using published clinical guidelines, articles, and physicians' heuristics and experiences. A rigorous inspection method was used to represent the clinical knowledge in the form of mind maps. In our study, the 2016 European Society of Cardiology guidelines and the 2017 American Heart Association/American College of Cardiology guidelines for the diagnosis and treatment of HF were selected to acquire and transform the knowledge. The mind map was then reviewed by the physicians and transformed into a formal representation of decision tree (DT) by knowledge engineers in review meetings with physicians. The initial DT was termed as clinical knowledge model (CKM), called expert-driven knowledge (EKD) and included 14 contributing factors (**Supplementary table 1**) and 4 possible outcomes: HF with reduced ejection fraction, HF with mid-range ejection fraction, HF with preserved ejection fraction, and not HF.

ML-drivenn rule generation
We used a white box algorithm to generate data driven rules in form of prediction model (PM) with a high accuracy based on the available datasets, and it is called ML-drivenn knowledge (MDK). The criteria for the selection of the final machine learning algorithm included the highest accuracy with a minimal set of decision paths in PM, which contain fewer conditional attributes.[13]

We applied machine learning algorithms such as DT, Random Forest, CHAID (Chi-squared Automatic Interaction Detection), J48, and CART (Classification and Regression Tree) on 600 patients for model training, and each algorithm provided a different accuracy **(Supplementary table 3)**. We used Rapid Miner and SPSS tools for analysis and for creating models to identify the most appropriate algorithm.

Hybrid knowledge: Hybrid knowledge is the merge of the CKM from the EDK approach and the PM from the MDK approach **(Supplementary figure 3)**, and ensures the final refined-clinical knowledge model (R-CKM) [13], to build a hybrid knowledge (HK), shown in **(Supplementary figure 1)**. In our previous work, we defined a 4-step validation criteria to merge the MDK and EDK. The R-CKM is thoroughly validated by the expert physicians. Our proposed Hybridization Algorithm has seven steps, which are described below.

Knowledge Hybridization Algorithm:

Step 1: Set the validation and conformance criteria C_v by physicians. The four criteria shown in following table.
Step 2: Select each path P_i from PM (till the all paths traversing).
Step 3: Select each c_i from criteria C_v (till the checking of all criterion).
Step 4: Check criterion c_i for each selected path P_i of PM for its conformance.
Step 5a: If criterion c_i is passed for path P_i then move to next criterion, and inspect path P_i and refine to P_j if required.
Step 5b: If criterion c_i is failed for path P_i then criterion c_i is checked for its priority, if it is primary then skip path. If it is not primary criterion then go to next criterion.
Step 6: using step 5a and 5b is check for each passed path and for each criterion, repetitively.
Step 7: Evolve R-CKM by adding P_j and updating the existing paths if required.

Following table describes the validation and merging criteria.

Criteria No.	Criteria	Priority	Primary	Remarks
1	$\{\forall P_i \in \text{PM} : \text{Accuracy}(P_i) > N\%\}$	1	Yes	• The domain expert assigns N, which represents the accuracy of PM based on the training data.<br>• Tradeoff : Higher accuracy setting produces an efficient model, but coverage of involving more patient features is limited and vice versa
2	$\{\forall P_i \in \text{PM} \land \forall P_j \in \text{CKM}: !\text{Conflict}(P_i, P_j)\}$	1	Yes	• Conflicts with guidelines; conflicting treatments must not be exist.<br>• Example : after surgery, chemo-induction has no meaning
3	$\{\forall P_i \in \text{PM} \land \exists P_j \in \text{CKM} : \text{Conform}(P_i, P_j) \text{ yields} \rightarrow P_i \in \text{R-CKM}\}$	2	No	• Decision path in PM conforming to any CKM path shall be part of R-CKM.
4	$\{\exists P_i \in \text{PM} \land \forall P_j \in \text{CKM} : !\text{Conform}(P_i, P_j) \text{ provides} \rightarrow \text{Evidence } (P_i) \text{ yields} \rightarrow P_i \in \text{R-CKM}\}$	3	No	• Decision path in PM not conforming to any path in CKM can be part of R-CKM only if:<br>• Sufficient evidence exists for effectiveness of the treatment.<br>• Evidence can be other standard clinical knowledge resources or local practices with a reasonable success ratio for the predicted treatment.

Knowledge transformation: The R-CKM was then transformed into a computer-executable format. We selected Health Level 7 standard representation of knowledge, called Arden Syntax Medical Logic Module (MLM), to share and disseminate the created knowledge among the diverse medical institutions. We designed and developed an Intelligent Knowledge Authoring Tool,[15] which transforms the R-CKM into shareable MLM and a computer-executable knowledge base. In this study of HF diagnosis, the R-CKM is demonstrated in **Supplementary figure 1**. All MLMs can execute individually or recursively by the developed MLM-based reasoner, and can provide the diagnosis decision based on the input (patient data).
Executable environment: We developed a CDSS-executable environment with a graphical user interface. The physicians enter the signs and symptoms, clinical history, physical examination, and other attributes, as mentioned in **Table 1**.

Supplementary Table 1. List of contributing factors in CKM

S. No.	Attribute Name	Attribute Description
1	Signs & Symptoms	Patient has some sign and symptom like, breathlessness, exercise tolerance, tiredness, ankle swelling, and nocturnal cough.
2	Clinical History	It checks the patient history such as coronary artery disease (CAD), arterial hypertension, exposition to cardio toxic drug/radiation, use of diuretics, orthopnea.
3	Physical Examination	In this category, physicians check rales, bilateral ankle edema, heart murmur, jugular venous dilatation, laterally displaced apical beat.
4	ECG Result	Noninvasive test to check how fast the heat beats, it may be normal or abnormal.
5	BNP Result	B-type natriuretic peptide (BNP) blood test measures the levels of the BNP hormone in patients' blood.
6	NT-proBNP Result	N-terminal pro-B-type natriuretic peptide level
7	Left Ventricular Ejection Fraction (LVEF)	It finds total amount of blood in the left ventricle is pumped out with each heartbeat.
8	Left Atrial Volume Index (LAVI)	Measure to evaluate the LA size
9	E/e'	Measure to evaluate the diastolic function
10	e' Septal	Measure to evaluate the diastolic function
11	Longitudinal strain	Measure to evaluate myocardial contractility
12	Tricuspid Regurgitation Velocity (TRV)	TRV has been shown to correlate with pulmonary artery systolic pressure (PASP) at rest (1–3) and with exercise (3–7).
13	Left Ventricular Mass Index (LVMI)	Measure to evaluate the LV size
14	Gender	The state of being male or female

Supplementary Table 2. Selected features by different machine learning algorithms as contributing factors

ML Algorithms	No. of Features	Sequence of Features
CART	4	a) LVEF, b) LAVI, c) LVMI, d) TRV
J48	7	a) LVEF, b) LAVI, c) TRV, d) ECG, e) E/e, f) LVMI, g) Clinical History
Random Forest	4	a) LAVI, b) LVMI, c) LVEF, d) Physical Exam
Decision Tree	3	a) LVEF, b) LAVI, c) Physical Exam
CHAID	4	a) LVEF, b) LAVI, c) LVMI, d) ECG

CART = The Classification and Regression Tree; CHAID = Chi-squared Automatic Interaction Detector; ECG = electrocardiography; LAVI = left atrial volume index; LVEF = left ventricular ejection fraction; LVMI = left ventricular mass index; TRV = tricuspid regurgitation velocity

Supplementary Table 3. Accuracy of machine learning algorithms in the provided dataset to select the final prediction model

Algorithms	Accuracy	Number of Rules	Number of Attributes	Ranking
CART	88.5%	5	4	0.5736
J48	84.7%	9	7	0.5549
Random Forest	83.63%	7	4	0.5438
Decision Tree	82.94	7	3	0.5388
CHAID	79.8%	7	4	0.5195

Supplementary Table 4. Confusion matrix of the Expert –Driven CDSS for diagnosis

	Predicted				% Correct	Sensitivity	Specificity
**Observed**	**HFrEF**	**HFmrEF**	**HFpEF**	**NOT HF**			
**HFrEF**	199	0	0	0	100%	0.76	1
**HFmrEF**	0	63	0	0	100%	0.51	1
**HFpEF**	0	1	187	40	82.05%	0.90	0.89
**NOT HF**	0	0	19	89	82.41%	0.68	0.95
**Percentage**	100%	98.43%	90.77%	68.99%	90%	0.71	0.96

Supplementary Table 5. Confusion matrix of the CART algorithm for prediction model generation

	Predicted				% Correct	Sensitivity	Specificity
**Observed**	**HFrEF**	**HFmrEF**	**HFpEF**	**NOT HF**			
**HFrEF**	0	0	199	0	100.0%	0.74	1
**HFmrEF**	63	0	0	0	100.0%	0.47	1
**HFpEF**	0	180	0	48	78.9%	0.89	0.87
**NOT HF**	0	21	0	87	80.6%	0.8	0.9
**Percentage**	100%	89.5%	100%	64.4%	88.5%	0.72	0.94

Supplementary Table 6. Confusion matrix of the Proposed Hybridization algorithm

	Predicted				% Correct	Sensitivity	Specificity
**Observed**	**HFrEF**	**HFmrEF**	**HFpEF**	**not HF**			
**HFrEF**	199	0	0	0	100%	0.95	1
**HFmrEF**	0	65	0	0	100%	0.86	1
**HFpEF**	0	0	227	1	99.56%	0.96	0.99
**not HF**	0	0	9	99	91.67%	0.99	0.98
**Percentage**	100%	100%	96.19%	99%	98.3%	0.94	0.99

Supplementary Table 7. Diagnostic accuracy according to patients' age

Age	Number of Patients	Correct Classified	False Classified	Concordance
20 - 40	27	26	1	96.29%
41 - 60	82	81	1	98.78%
61 - 80	317	312	5	98.42%
81 - 100	174	171	3	98.28%

Supplementary Figures

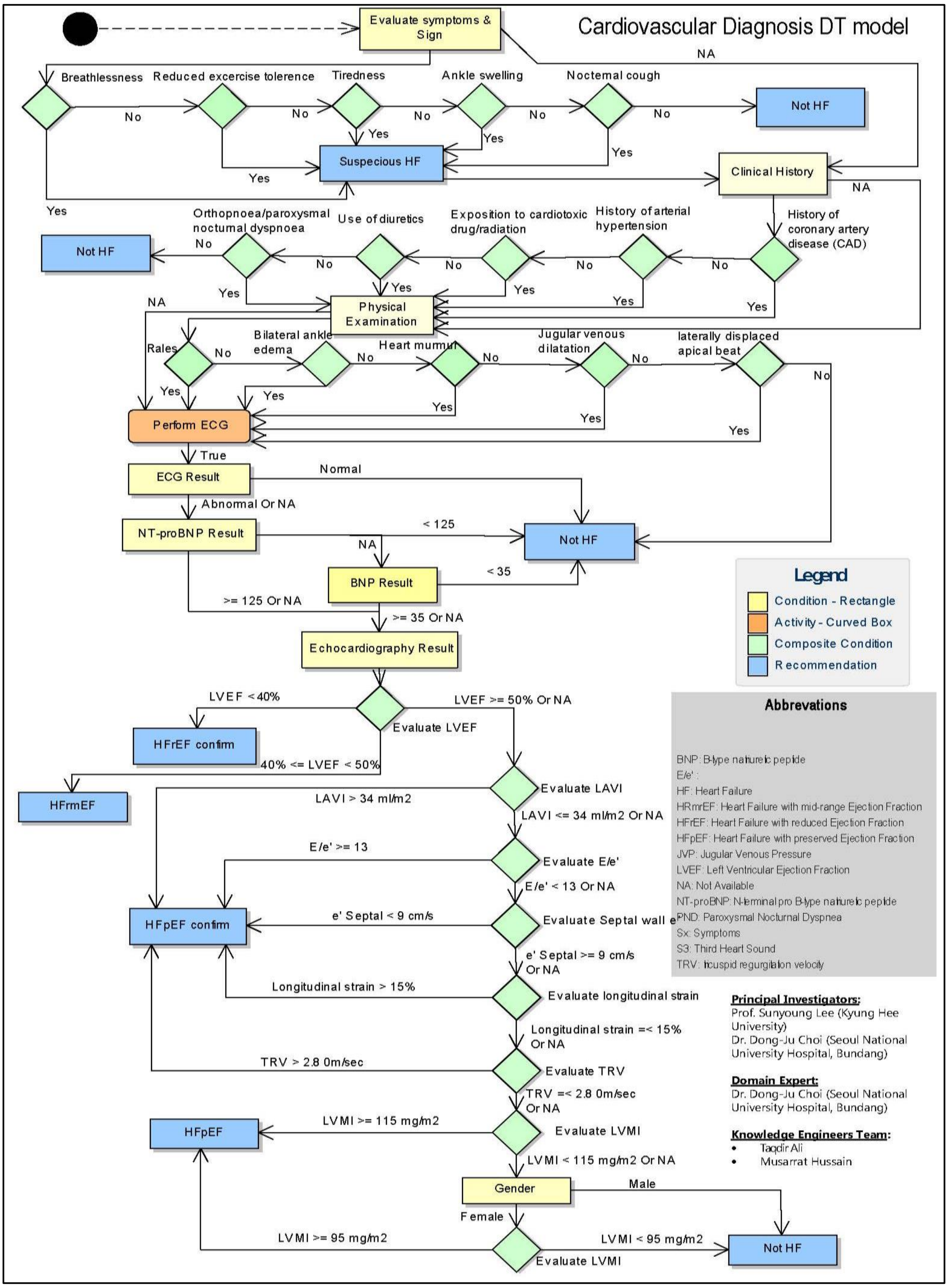

Supplementary Figure 1: Refined-clinical knowledge model (CKM), made using hybridization of expert-driven CKM and ML-driven prediction model.

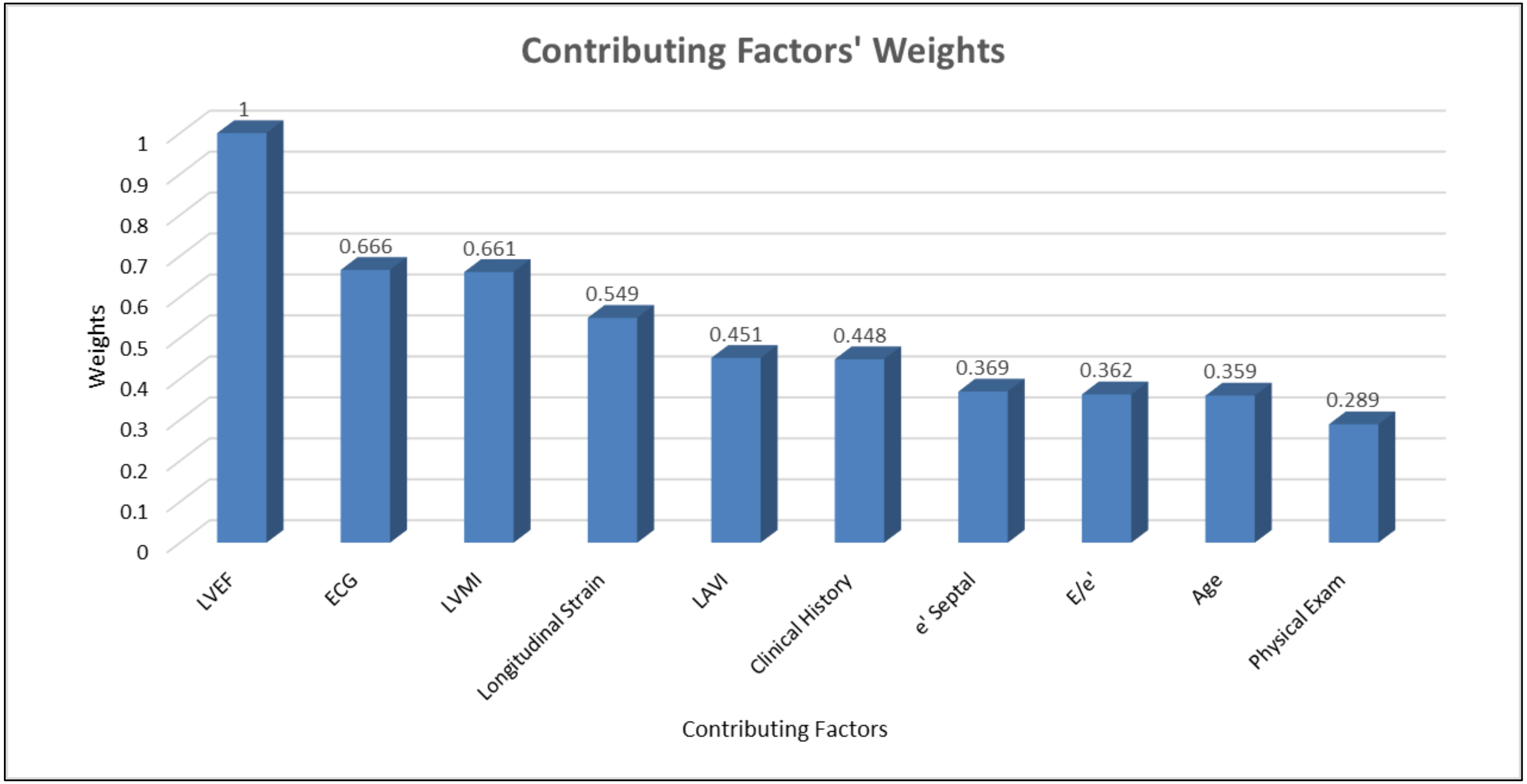

Supplementary Figure 2: Contributing factors selected by machine learning during the ML-drivenn approach.

ECG = electrocardiography; LAVI = left atrial volume index; LVEF = left ventricular ejection fraction; LVMI = left ventricular mass index

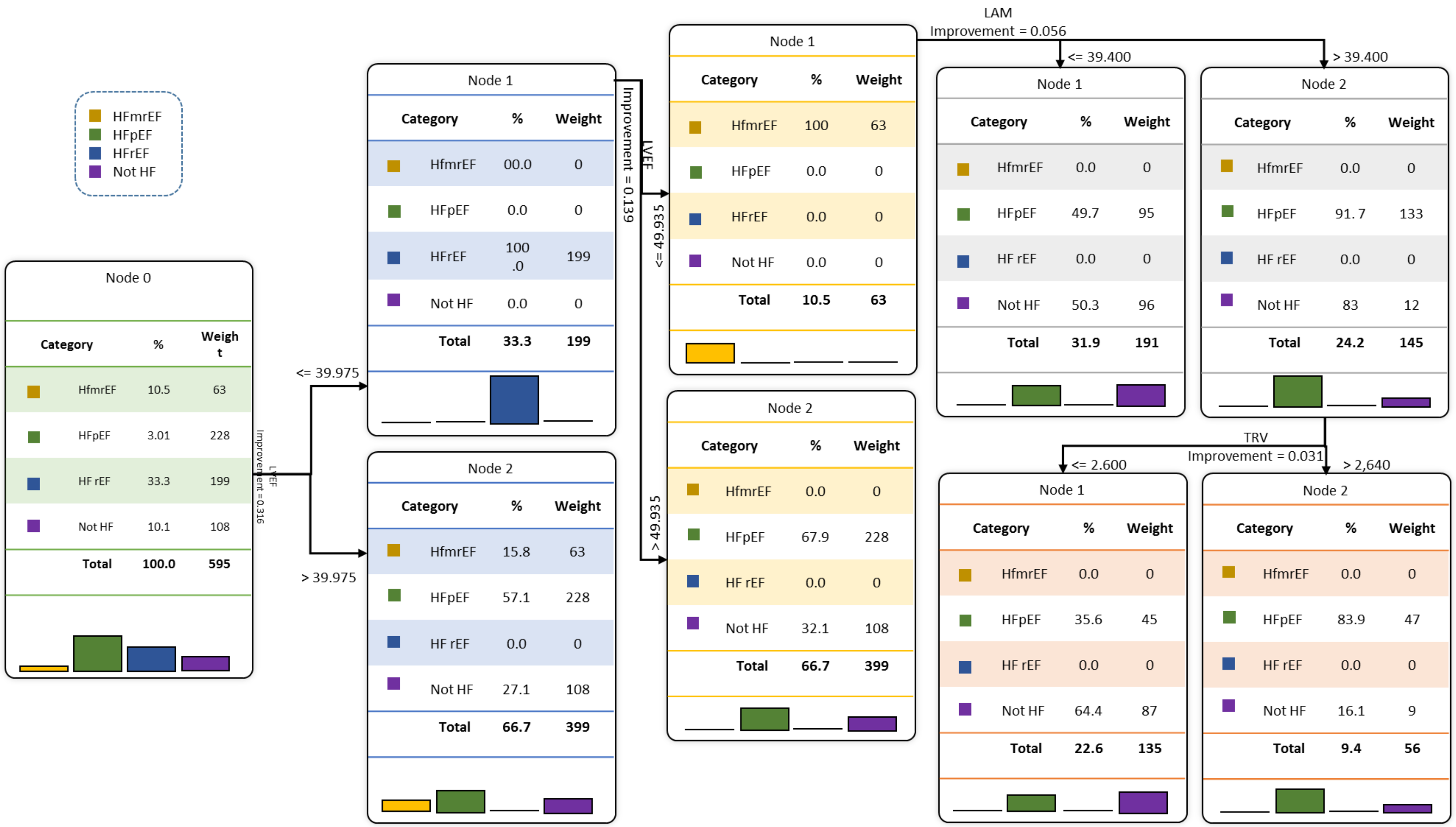

Supplementary Figure 3: Final selected prediction model generated by the CART machine learning algorithm with the highest ranking value (0.5736).

HFmrEF= heart failure with mid-range ejection fraction; HFpEF = heart failure with preserved ejection fraction; HFrEF = heart failure with reduced ejection fraction; LAVI = left atrial volume index; LVEF = left ventricular ejection fraction; LVMI = left ventricular mass index; TRV = tricuspid regurgitation velocity

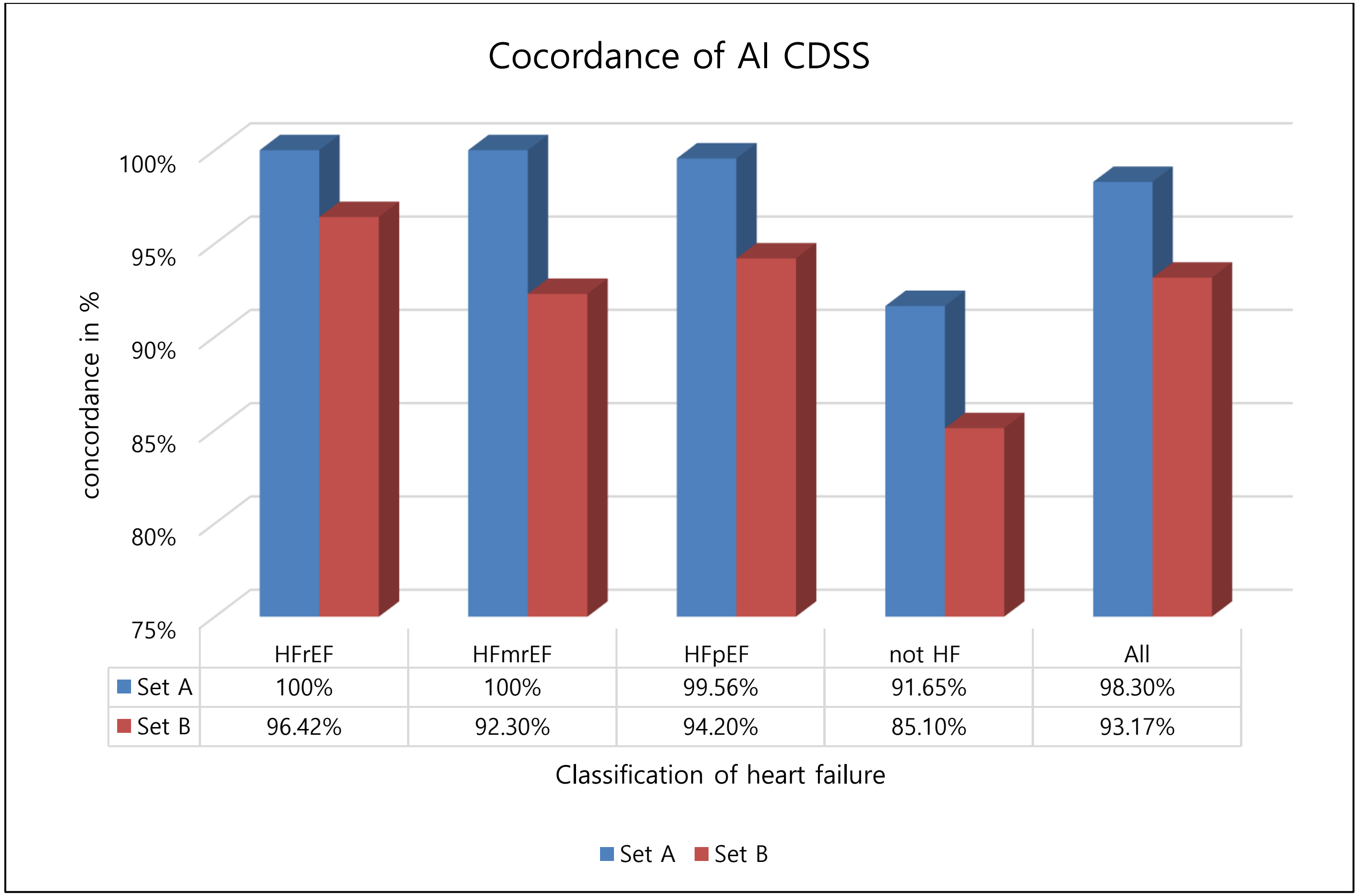

Supplementary Figure 4: Concordance rate comparison based on set A (includes all echocardiography parameters) and set B (includes only LVEF, LAVI, and LVMI).

HFmrEF= heart failure with mid-range ejection fraction; HFpEF = heart failure with preserved ejection fraction; HFrEF = heart failure with reduced ejection fraction
